# Assessing the impact of medically assisted reproduction on autism spectrum disorder risk

**DOI:** 10.1007/s10815-024-03180-z

**Published:** 2024-06-26

**Authors:** Omri Zamstein, Tamar Wainstock, Gil Gutvirtz, Eyal Sheiner

**Affiliations:** 1 The Obstetrics and Gynecology Division, Soroka University Medical Center, Ben-Gurion University of the Negev, Be’er-Sheva, Israel; 2 Faculty of Health Sciences, School of Public Health, Ben-Gurion University of the Negev, Be’er-Sheva, Israel

**Keywords:** Ovulation induction, In vitro fertilization, Epigenetics, Autism spectrum disorder

## Abstract

**Purpose:**

Techniques of medically assisted reproduction interact with the embryo at crucial developmental stages, yet their impact on the fetus and subsequent child’s health remains unclear. Given rising infertility rates and more frequent use of fertility treatments, we aimed to investigate if these methods heighten the risk of autism spectrum disorder (ASD) in children.

**Methods:**

A population-based cohort study was conducted at Soroka University Medical Center, a tertiary referral hospital, encompassing singleton births. The incidence of ASD in offspring, incorporating either hospital or community-based diagnoses, was compared in relation to the conception method. To examine the cumulative incidence of ASD, a Kaplan–Meier survival curve was utilized. Cox proportional hazards model was employed to adjust for confounders.

**Results:**

Among 115,081 pregnancies, 0.5% involved ovulation induction (OI) and 1.7% in vitro fertilization (IVF), with the rest conceived naturally. Fertility treatments were more common in older patients and linked to more diabetes, hypertensive disorders, preterm, and cesarean deliveries. Out of 767 ASD diagnoses, offspring from OI and IVF had higher initial ASD rates (2.1% and 1.3%) than natural conceptions (0.6%). In a Cox model accounting for maternal age, ethnicity, and gender, neither OI nor IVF was significantly associated with ASD. The adjusted hazard ratios were 0.83 (95% *CI* 0.48–1.43) for OI and 1.34 (95% *CI* 0.91–1.99) for IVF. When considering fertility treatments combined, the association with ASD remained non-significant (*aHR* 1.11, 95% *CI* 0.80–1.54, *p* = 0.52).

**Conclusion:**

Fertility treatments, including OI and IVF, do not exhibit a significant association with heightened ASD risk in offspring.

## Introduction

Though the exact mechanisms driving autism spectrum disorder (ASD) development are not fully understood, the commonly held view is that genetic factors influencing brain development, activated in response to environmental exposures, play a significant role [1]. Given the intricate and vulnerable process of in utero brain development [2], numerous obstetric conditions have been linked to an increased risk of ASD. These include pregnancy at an advanced age, infections during pregnancy, diabetes mellitus, hypertension, preterm delivery, and low birth weight [3–7]. Yet, few have examined the potential impacts of factors introduced during the periconceptional period, spanning the time preceding, encompassing, and immediately following conception [8, 9].

Infertility rates, the utilization of fertility treatments, and the efficacy of medically assisted reproduction techniques are all on the rise [10–12]. It is estimated that in developed countries, assisted reproductive technologies (ART) account for up to 5% of live births [11, 13]. Commonly employed techniques to address infertility include ovulation induction (OI) and in vitro fertilization (IVF), both involving the administration of medications to facilitate oocyte development. In the context of IVF, the process additionally involves oocyte retrieval, fertilization, and subsequently transfer of embryos to the uterus [14]. These procedures occur during the critical periconceptional time, shortly after which fetal brain development commences [2]. In the later stages of gestation, these pregnancies are also more susceptible to complications, which can pose a risk to the fetus [15, 16].

Previous studies examining the neurodevelopmental outcomes of offspring following fertility treatments have yielded inconsistent findings. While some studies indicated an elevated likelihood of ASD in offspring conceived by assisted reproduction, others limited this heightened risk to offspring of multifetal gestations [17, 18] or attributed it to the intensified developmental monitoring among children conceived through ART [19]. A recent study concluded that despite the association between infertility and ASD, fertility treatments did not introduce any additional risk compared to subfertility alone [20]. With increasing infertility rates and the growing use of fertility treatments, our objective was to further examine the links between reproductive techniques, obstetric characteristics, and childhood ASD risk.

## Materials and methods

A retrospective cohort study conducted at Soroka University Medical Center (SUMC) from 2005 to 2021 included deliveries of Clalit Health Services (CHS)-insured patients, the largest health maintenance organization in Israel. With healthcare services universally free in Israel and the similarities between patients covered by CHS and the general population [21], the study population is reflective of the entire population of southern Israel. This study received approval from the Institutional Review Board (IRB: 0357–19-SOR), in compliance with the Helsinki Declaration of Ethical Standards.

Study groups were categorized based on the method of conception: pregnancies achieved through OI using gonadotropin therapy; those resulting from IVF, which included techniques such as autologous or donor oocyte use, sperm insemination, or intracytoplasmic sperm injection (ICSI), with or without preimplantation genetic testing; and naturally conceived pregnancies. Initially, we evaluated obstetrical and perinatal outcomes using the SUMC perinatal database, which captures information recorded shortly after each delivery by attending obstetricians and reviewed by medical secretaries. Subsequently, we compared the likelihood of ASD diagnoses in offspring during childhood based on the mode of conception. ASD cases were identified by extracting diagnoses coded under ICD-9 from CHS outpatient clinic records or hospital records. It is important to note that screening and diagnostic evaluations for ASD are universally covered by insurance, facilitating comprehensive data collection and removing financial barriers to accurate reporting. The diagnosis of ASD is clinically confirmed in children who fulfill the criteria specified in the *Diagnostic and Statistical Manual of Mental Disorders, Fifth Edition* (DSM-5) [22]. According to national guidelines, the diagnosis must be a collaborative decision involving a clinical psychologist and a physician specialized in developmental disorders, neurology, or child psychiatry. The diagnostic process also typically includes assessments from speech therapists and occupational therapists, who evaluate the child’s language abilities, sensory sensitivities, and other relevant characteristics to thoroughly understand their condition and needs [23].

**Fig. 1:**
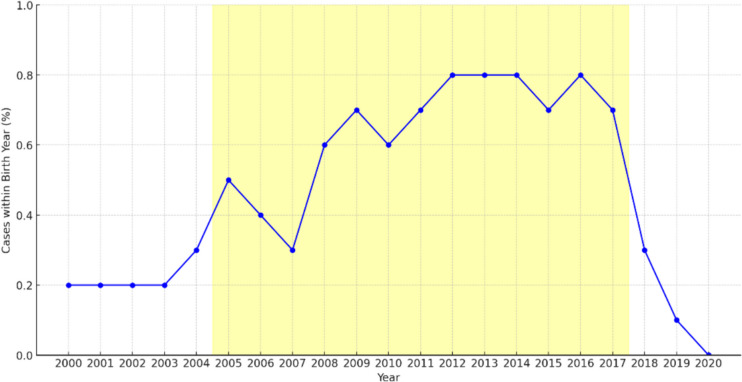
Incidence of autism spectrum disorder diagnoses by birth year, 2000–2020: highlighting increased incidence from 2005 to 2017

After preliminary analysis, only births occurring between the years 2005 and 2017 were considered. This timeframe was chosen to account for potential low ASD awareness before the year 2005 and to ensure that offspring born after 2017, who might not have been diagnosed by the study’s end in 2021, were excluded (Fig. 1) [24, 25]. This is supported by evidence that while ASD can be diagnosed as early as 2 to 3 years, many children often receive a definitive diagnosis later [26].

### Statistical analyses

SPSS for Windows (Version 29.0; IBM, Chicago, IL, USA) was used for statistical analysis. Categorical variables were described by frequencies, and normally distributed continuous variables were presented using mean and standard deviation. Differences between the groups were examined through the chi-square test for categorical variables and the unpaired *t*-test for continuous variables, as appropriate. Cases of perinatal mortality were excluded from the long-term analysis. Kaplan–Meier survival curves were built to assess the cumulative incidence of ASD diagnoses during childhood, and the Cox–Mantel log-rank test was employed to discern differences between the groups. Finally, we used Cox regression analyses to compare morbidity rates, adjusting for confounders that were identified as clinically relevant and accounting for multiple pregnancies in the same patient. Analyses were two-sided, with *p*-values < 0.05 considered significant.

## Results

Our study initially included 395,408 deliveries. We excluded 15,150 cases (3.8%) of multiple gestations and 23,902 cases (6.3% of the remainder) with significant congenital anomalies, primarily malformations of the central nervous system and chromosomal abnormalities, to mitigate potential confounding from these unique conditions [27]. Further, 123,880 deliveries (34.8% of the remainder) involving non-Clalit HMO patients were removed to ensure consistent healthcare records. Additionally, we confined our analysis to a defined study period, excluding another 117,395 cases (50.3% of the remainder), leading to a final cohort of 115,081 deliveries.

Of these 115,081 deliveries, 609 (0.5%) resulted from OI and 1991 (1.7%) from IVF, with the remainder occurring spontaneously. Generally, pregnancies following fertility treatments (OI or IVF) were more prevalent among older patients, with over half of them undergoing their first childbirth. These cases showed a significantly higher prevalence of diabetes mellitus and hypertension (*p* < 0.001; Table 1) and were more likely to be labor-induced and delivered at an earlier gestational age (*p* < 0.001 for all; Table 2). Both OI and IVF pregnancies had higher rates of cesarean deliveries (*CD*: 32.7% and 36.9%, respectively, vs. 14.7% for natural conception, *p* < 0.001). Birthweight was 100–200 g lower among pregnancies following fertility treatments, resulting in a higher proportion classified as low birth weight.

**Table 1:** Maternal characteristics by mode of conception

Characteristic	Ovulation induction (*n* = 609, 0.5%)	In vitro fertilization (*n* = 1991, 1.7%)	Natural conception (*n* = 112,481, 97.7%)	*p*-value
Maternal age (mean ± SD)	30.4 ± 5.2	32.5 ± 5.6	28.1 ± 5.7	< 0.001
Primiparity (%)	55.3	50.7	24.3	< 0.001
Ethnicity (%)				< 0.001
Bedouin	30.9	28.2	61.8	
Jewish	69.1	71.8	38.2	
Diabetes mellitus during pregnancy^a^ (%)	12.6	11.7	3.9	< 0.001
Hypertensive disorders during pregnancy^b^ (%)	8.4	8.0	3.8	< 0.001

^a^Includes pregestational and gestational diabetes mellitus

^b^Includes chronic hypertension and pregnancy-induced hypertensive disorders

**Table 2:** Obstetrical outcomes by mode of conception

Outcome	Ovulation induction (*n* = 609, 0.5%)	In vitro fertilization (*n* = 1991, 1.7%)	Natural conception (*n* = 112,481, 97.7%)	*p*-value
Mean gestational age (weeks ± SD)	38.4 ± 2.0	38.1 ± 2.3	39.0 ± 1.9	< 0.001
Preterm delivery (< 37 weeks, %)	11.0	11.2	6.8	< 0.001
FGR^a^ (%)	3.3	3.0	2.0	< 0.001
Induction of labor (%)	32.7	27.8	18.1	< 0.001
Non-reassuring fetal heart rate patterns (%)	9.9	9.6	6.2	< 0.001
Cesarean delivery (%)	32.7	36.9	14.7	< 0.001
5-min Apgar < 7 (%)	1.0	1.1	0.6	< 0.001
Cord blood pH < 7.0 (%)	0.0	0.7	0.3	0.700
Birthweight, g (mean ± SD)	3079 ± 569	3068 ± 575	3187 ± 513	< 0.001
Birth weight < 2500 g (%)	13.3	11.6	7.1	< 0.001
Offspring gender				0.105
Female (% all)	51.7	51.3	49.3	
Male (% all)	48.3	48.7	50.7	
Perinatal mortality (cases, %)	5 (0.8)	26 (1.3)	849 (0.8)	0.039

^a^Fetal growth restriction: estimated fetal weight or abdominal circumference < 10th percentile for gestational age

**Fig. 2:**
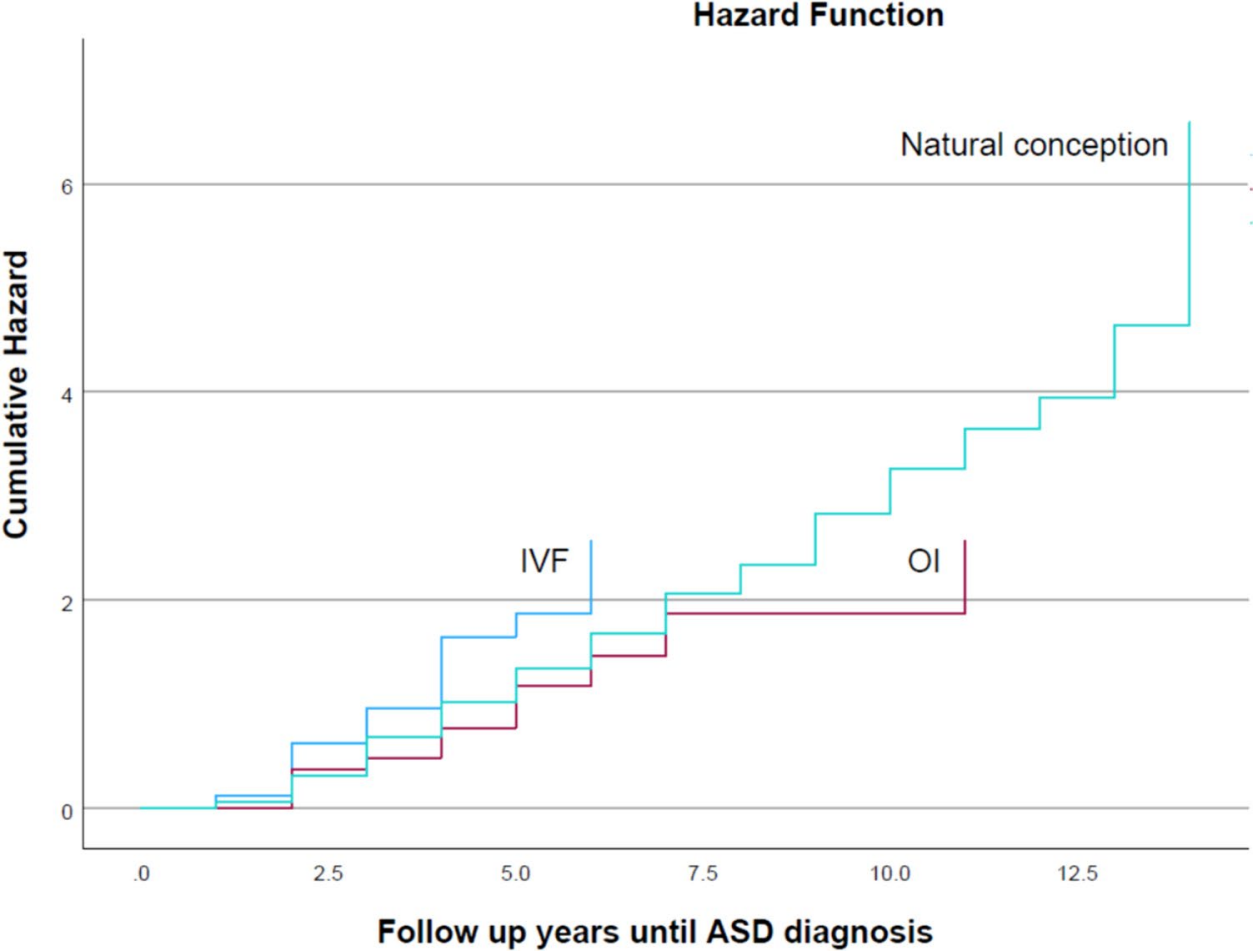
Cumulative incidence of autism spectrum disorder (ASD) diagnoses in offspring according to conception method (log-rank *p*-value = 0.136)

During the follow-up, ASD developed in 767 children within the cohort: 13 (2.1%) from OI, 26 (1.3%) from IVF, and 728 (0.6%) from the natural conception group. Median follow-up times were 6 years for the IVF group (*IQR*: 4–10 years) and 8 years for both the OI (*IQR*: 5–12 years) and natural conception groups (*IQR*: 5–11 years). The rates of ASD per 1000 person-years were 2.45 in the OI group, 1.82 in the IVF group, and 0.79 in the natural conception group. Fertility treatments (OI and IVF combined) were associated with a significantly higher ASD prevalence than natural conceptions, evidenced by an odds ratio of 2.34 (95% *CI* 1.69–3.23, *p* < 0.001; Table 3). The hazard curve (Fig. 2) displays the cumulative ASD incidence across three groups, revealing no significant differences between fertility treatments (OI or IVF) and natural conception in ASD rates (log-rank *p*-value = 0.136).

**Table 3:** The association between mode of conception and autism spectrum disorder (ASD) in the offspring

	Ovulation induction (*n* = 609, 0.5%)	In vitro fertilization (*n* = 1991, 1.7%)	Natural conception (*n* = 112,481, 97.7%)	*p*-value
ASD (% total)	13 (2.1%)	26 (1.3%)	728 (0.6%)	< 0.001
Odds ratio	2.34 (95% *CI* 1.69–3.23)		1 (reference)	< 0.001
Risk difference	1.5%	0.7%	-	
Adjusted HR^a^	0.83 (95% *CI* 0.48–1.43, *p* = 0.499)	1.34 (95% *CI* 0.91–1.99, *p* = 0.142)	1 (reference)	
Adjusted HR^a^	1.11 (95% *CI* 0.80–1.54, *p* = 0.52)		1 (reference)	

^a^Adjusted for maternal age, ethnicity, and fetal gender

In a Cox proportional hazards model accounting for maternal age, ethnicity, and offspring gender, neither OI nor IVF was significantly associated with childhood ASD. The adjusted hazard ratios (aHR) were 0.83 (95% *CI* 0.48–1.43, *p* = 0.499) for OI and 1.34 (95% *CI* 0.91–1.99, *p* = 0.142) for IVF (Table 3). When considering fertility treatments combined, the association with ASD remained nonsignificant (*aHR* 1.11, 95% *CI* 0.80–1.54, *p* = 0.52).

## Discussion

In this cohort study involving over 115,000 singleton pregnancies, those resulting from fertility treatments (OI or IVF) exhibited high-risk pregnancy features and a tendency toward earlier deliveries and lower birthweights. Initial univariate analysis suggested increased ASD rates in the OI group; however, after adjusting for maternal age, ethnicity, and offspring gender, neither OI nor IVF were found to be independently associated with ASD in children.

Regarding pregnancy and birth outcomes following fertility treatments, our findings are consistent with the current body of literature, indicating an increased likelihood of placental-related complications, preterm birth, CD, and low birth weight [28]. However, the absolute increase in risk is generally viewed as modest, with most of these pregnancies yielding normal perinatal outcomes [29, 30].

Although current evidence in the literature is inconclusive, our long-term results align with four previous studies that focused on ASD risk among singletons, adjusting for confounding factors [18, 31–33]. A meta-analysis that included these studies calculated a pooled aHR of 0.96 (95% *CI* 0.82–1.13, *p* = 0.654) [17]. Another large population-based study in Denmark similarly found no significant increase in the risk of ASD in children born after assisted conception when adjusting for confounders like maternal age and parity. Although there was an initial crude hazard rate ratio of 1.25 (95% *CI* 1.09–1.43), this association disappeared after adjustments (1.13; 95% *CI* 0.97–1.31), indicating no overall risk of ASD linked to fertility treatments [34]. However, our results contrast with other studies showing a small increased risk of ASD among offspring from the broader ART population (*aHR* 1.11, 95% *CI* 1.03–1.19, *p* = 0.009). This discrepancy highlights the importance of considering factors such as multiple pregnancies, though other methodological aspects, like the inclusion of case–control studies or not differentiating fertility treatment types, may have affected the results. Velez et al.’s recent cohort study also identified an attenuation of ASD risk in IVF offspring after restricting to singleton births (*aHR* 1.03, 95% *CI* 0.91–1.17). Contrary to our results, however, an elevated risk persisted for OI and IUI (*aHR* 1.15, 95% *CI* 1.03–1.29) and was also present among children of infertile couples who did not undergo fertility treatments (*aHR* 1.20, 95% *CI* 1.15–1.25) [20].

Both treatment- and parental-related factors could potentially influence the development and future health of embryos achieved through fertility treatments. IVF, for example, involves interventions like exposure to hormonal, cryopreservant, and culture-media agents, as well as manipulation of gametes and embryos, potentially altering gene expression patterns (epigenetic modifications) or compromising the embryo mechanically [35, 36]. Apart from specific imprinting disorders such as Beckwith-Wiedemann syndrome that have been previously linked with ART [37], evidence suggest that epigenic alterations following ART may occur throughout the genome, even in seemingly normal offspring [38]. Correct epigenetic programming is important for accurate development, as it controls cell cleavage, cell determination, and expression of early embryonic genes, including those related to neural cells [36]. Although there is a suspicion that aberrant gene expression contributes to neurodevelopmental disorders like ASD [39], the specific relationship between certain imprinted loci and the nuances of cognitive and behavioral functions remains unclear [40]. Additionally, the phenotypic impact of epigenetic modifications is uncertain, as gene transcription may be influenced by different mechanisms as well [41].

Parental factors potentially influencing offspring health include the advanced age of IVF patients (with a reported 30% increased risk of having offspring with ASD over the age of 35 [3]), maternal comorbidities [42], and the emotional stress associated with infertility [43]. The socioeconomic status of infertile couples often correlates with their awareness of child development and access to healthcare [44]. It is also plausible that underlying factors contributing to infertility could be associated with neuropsychiatric disorders in children [20]. This could arise either from elevated androgen levels during fetal growth, as seen in polycystic ovary syndrome, from the inflammatory environment often linked to endometriosis and obesity, or from genetic variations in the sperm of oligospermic males [32, 36, 45].

Our study carries limitations, notably the lack of control for other potential confounding variables such as treatment protocols, paternal age, additional environmental exposures that might affect brain development [25], and education level. Additionally, while ASD diagnoses were primarily sourced from SUMC’s inpatient and CHS-affiliated outpatient clinics, some children may have received care elsewhere, a factor that likely impacted both the study and comparison groups similarly and did not significantly alter the results. We also lacked information on whether ICSI was employed during IVF cycles. While ICSI is primarily used for male factor infertility, its application has significantly expanded globally over the past decades to include cycles without male factor infertility [46]. Its use, especially when combined with suboptimal sperm, is suspected to independently contribute to genetic alterations in the progeny [29]. The challenges of understanding the unique profiles of infertile patients, the effects of treatment, the complex nature of ASD, and its underlying biological pathways, combined with the inherent limitations of observational studies, hinder the ability to definitively establish or refute a causal relationship.

The main strengths of the study include high follow-up rates and strong generalizability, achieved through a well-balanced sample representative of the population [47]. In Israel, fertility treatments including IVF are readily accessible and fully covered by the National Health Insurance Law to those who require them for medical reasons, irrespective of their socioeconomic status [48]. Also, including ASD diagnoses from both hospital and outpatient settings adds to the reliability of our findings.

In summary, this research sheds light on the health outcomes of children conceived via fertility treatments, emphasizing the complex interaction between treatment and parental factors on embryonic health. Our findings suggest that fertility treatments alone do not markedly elevate the risk of ASD, highlighting the need for further research to fully understand the wider implications of these procedures. Distinguishing the impact of fertility treatments from parental factors is essential for enabling healthcare providers and prospective parents to make well-informed decisions and to customize postnatal care and follow-up strategies accordingly.

## Data Availability

The data supporting this article can be provided upon a reasonable request to the corresponding author, subject to IRB approval and conditions.

## References

[1] Lai MC, Lombardo MV, Baron-Cohen S. Autism. Lancet (London, England). 2014;383(9920):896–910. 24074734 10.1016/S0140-6736(13)61539-1

[2] Connors SL, Levitt P, Matthews SG, Slotkin TA, Johnston MV, Kinney HC, et al. Fetal mechanisms in neurodevelopmental disorders. Pediatr Neurol. 2008;38(3):163–76. 18279750 10.1016/j.pediatrneurol.2007.10.009

[3] Sandin S, Hultman CM, Kolevzon A, Gross R, MacCabe JH, Reichenberg A. Advancing maternal age is associated with increasing risk for autism: a review and meta-analysis. J Am Acad Child Adolesc Psychiatry. 2012;51(5):477-86.e1. 22525954 10.1016/j.jaac.2012.02.018

[4] Xiang AH, Wang X, Martinez MP, Walthall JC, Curry ES, Page K, et al. Association of maternal diabetes with autism in offspring. JAMA. 2015;313(14):1425–34. 25871668 10.1001/jama.2015.2707

[5] Amaral DG. Examining the causes of autism. Cerebrum. 2017;2017:1 PMC5501015 28698772

[6] Crump C, Sundquist J, Sundquist K. Preterm or early term birth and risk of autism. Pediatrics. 2021;148(3):e2020032300. 10.1542/peds.2020-032300. 10.1542/peds.2020-032300 PMC9809198 34380775

[7] Ma X, Zhang J, Su Y, Lu H, Li J, Wang L, et al. Association of birth weight with risk of autism: a systematic review and meta-analysis. Res Autism Spectrum Disorders. 2022;92:101934.

[8] Louis GMB, Cooney MA, Lynch CD, Handal A. Periconception window: advising the pregnancy-planning couple. Fertility and Sterility. 2008;89(2 Supplement):e119–21. 18308052 10.1016/j.fertnstert.2007.12.043 PMC2527691

[9] Steegers-Theunissen RPM, Twigt J, Pestinger V, Sinclair KD. The periconceptional period, reproduction and long-term health of offspring: the importance of one-carbon metabolism. Hum Reprod Update. 2013;19(6):640–55. 23959022 10.1093/humupd/dmt041

[10] Sun H, Gong TT, Jiang YT, Zhang S, Zhao YH, Wu QJ. Global, regional, and national prevalence and disability-adjusted life-years for infertility in 195 countries and territories, 1990–2017: results from a global burden of disease study, 2017. Aging (Albany NY). 2019;11(23):10952–91. 31790362 10.18632/aging.102497 PMC6932903

[11] Sunderam S, Kissin DM, Zhang Y, Jewett A, Boulet SL, Warner L, et al. Assisted reproductive technology surveillance - United States, 2018. Morbidity and mortality weekly report Surveillance summaries (Washington, DC : 2002). 2022;71(4):1–19. 10.15585/mmwr.ss7104a1 PMC8865855 35176012

[12] Wade JJ, MacLachlan V, Kovacs G. The success rate of IVF has significantly improved over the last decade. Aust N Z J Obstet Gynaecol. 2015;55(5):473–6. 26174052 10.1111/ajo.12356

[13] Israel HMo. In Vitro fertilization treatments 1990–2021 Jerusalem: data division. 2023. [Available from: https://www.gov.il/BlobFolder/reports/ivf-reports/he/files_publications_info_unit_IVF1990-2021.pdf. Accessed 31 Dec 2023.

[14] Fertility problems: assessment and treatment. London: National institute for health and care excellence (NICE); 2017 32134604

[15] Ghidini A, Gandhi M, McCoy J, Kuller JA. Society for Maternal-Fetal Medicine Consult Series #60: management of pregnancies resulting from in vitro fertilization. Am J Obstet Gynecol. 2022;226(3):B2-b12. 34736912 10.1016/j.ajog.2021.11.001 PMC13231394

[16] Silberstein T, Levy A, Harlev A, Saphier O, Sheiner E. Perinatal outcome of pregnancies following in vitro fertilization and ovulation induction. J Mater-Fetal Neonatal Med. 2014;27(13):1316–9. 10.3109/14767058.2013.856415 24175873

[17] Andreadou MT, Katsaras GN, Talimtzi P, Doxani C, Zintzaras E, Stefanidis I. Association of assisted reproductive technology with autism spectrum disorder in the offspring: an updated systematic review and meta-analysis. Eur J Pediatr. 2021;180(9):2741–55. 34279715 10.1007/s00431-021-04187-9

[18] Sandin S, Nygren KG, Iliadou A, Hultman CM, Reichenberg A. Autism and mental retardation among offspring born after in vitro fertilization. JAMA. 2013;310(1):75–84. 23821091 10.1001/jama.2013.7222

[19] Schieve LA, Fountain C, Boulet SL, Yeargin-Allsopp M, Kissin DM, Jamieson DJ, et al. Does autism diagnosis age or symptom severity differ among children according to whether assisted reproductive technology was used to achieve pregnancy? J Autism Dev Disord. 2015;45(9):2991–3003. 25997596 10.1007/s10803-015-2462-1 PMC4553150

[20] Velez MP, Dayan N, Shellenberger J, Pudwell J, Kapoor D, Vigod SN, et al. Infertility and risk of autism spectrum disorder in children. JAMA Netw Open. 2023;6(11):e2343954. 37983032 10.1001/jamanetworkopen.2023.43954 PMC10660172

[21] Wainstock T, Sergienko R, Orenshtein S, Sheiner E. Factors associated with COVID-19 vaccination likelihood during pregnancy. Int J Gynaecol Obstetrics. 2023;161(2):478–84. 10.1002/ijgo.14680 36651802

[22] Lord C, Bishop SL. Recent advances in autism research as reflected in DSM-5 criteria for autism spectrum disorder. Annu Rev Clin Psychol. 2015;11:53–70. 25581244 10.1146/annurev-clinpsy-032814-112745

[23] Israel MoHo. Autism spectrum disorder: diagnosis, legal rights, and educational programs. 2022. [Available from: https://www.gov.il/en/pages/mental-health-autism?chapterIndex=2. Accessed 31 Dec 2023.

[24] Siu AL, Bibbins-Domingo K, Grossman DC, Baumann LC, Davidson KW, Ebell M, et al. Screening for autism spectrum disorder in young children: US Preventive Services Task Force Recommendation Statement. JAMA. 2016;315(7):691–6. 26881372 10.1001/jama.2016.0018

[25] Hyman SL, Levy SE, Myers SM. council on children with disabilities, section on developmental and behavioral pediatrics. Identification, evaluation, and management of children with autism spectrum disorder. Pediatrics. 2020;145(1):e20193447. 10.1542/peds.2019-3447. 10.1542/peds.2019-3447 31843864

[26] van 't Hof M, Tisseur C, van Berckelear-Onnes I, van Nieuwenhuyzen A, Daniels AM, Deen M, et al. Age at autism spectrum disorder diagnosis: a systematic review and meta-analysis from 2012 to 2019. Autism. 2021;25(4):862-73 10.1177/1362361320971107 33213190

[27] Multifetal gestations: twin, triplet, and higher-order multifetal pregnancies: ACOG Practice Bulletin, Number 231. Obstetrics and gynecology. 2021;137(6):e145-e62. 10.1097/AOG.0000000000004397 34011891

[28] Qin J, Liu X, Sheng X, Wang H, Gao S. Assisted reproductive technology and the risk of pregnancy-related complications and adverse pregnancy outcomes in singleton pregnancies: a meta-analysis of cohort studies. Fertility and Sterility. 2016;105(1):73-85.e1-6. 26453266 10.1016/j.fertnstert.2015.09.007

[29] Committee Opinion No 671: perinatal risks associated with assisted reproductive technology. Obstetrics and gynecology. 2016;128(3):e61–8. 10.1097/AOG.0000000000001643 27548556

[30] Henningsen AA, Gissler M, Skjaerven R, Bergh C, Tiitinen A, Romundstad LB, et al. Trends in perinatal health after assisted reproduction: a Nordic study from the CoNARTaS group. Human Reproduction (Oxford, England). 2015;30(3):710–6. 25605701 10.1093/humrep/deu345

[31] Lehti V, Brown AS, Gissler M, Rihko M, Suominen A, Sourander A. Autism spectrum disorders in IVF children: a national case-control study in Finland. Human Reproduction (Oxford, England). 2013;28(3):812–8. 23293220 10.1093/humrep/des430 PMC3708520

[32] Schieve LA, Drews-Botsch C, Harris S, Newschaffer C, Daniels J, DiGuiseppi C, et al. Maternal and paternal infertility disorders and treatments and autism spectrum disorder: findings from the study to explore early development. J Autism Dev Disord. 2017;47(12):3994–4005. 28900768 10.1007/s10803-017-3283-1 PMC5804352

[33] Klemetti R, Sevón T, Gissler M, Hemminki E. Health of children born as a result of in vitro fertilization. Pediatrics. 2006;118(5):1819–27. 17079550 10.1542/peds.2006-0735

[34] Hvidtjørn D, Grove J, Schendel D, Schieve LA, Sværke C, Ernst E, et al. Risk of autism spectrum disorders in children born after assisted conception: a population-based follow-up study. J Epidemiol Community Health. 2011;65(6):497–502. 20584728 10.1136/jech.2009.093823

[35] The role of assisted hatching in in vitro fertilization: a guideline. Fertility and sterility. 2022;117(6):1177–82. 10.1016/j.fertnstert.2022.02.020 35618358

[36] De Rycke M, Liebaers I, Van Steirteghem A. Epigenetic risks related to assisted reproductive technologies: risk analysis and epigenetic inheritance. Human Reproduction (Oxford, England). 2002;17(10):2487–94. 12351517 10.1093/humrep/17.10.2487

[37] Vermeiden JP, Bernardus RE. Are imprinting disorders more prevalent after human in vitro fertilization or intracytoplasmic sperm injection? Fertil Steril. 2013;99(3):642–51. 23714438 10.1016/j.fertnstert.2013.01.125

[38] Batcheller A, Cardozo E, Maguire M, DeCherney AH, Segars JH. Are there subtle genome-wide epigenetic alterations in normal offspring conceived by assisted reproductive technologies? Fertil Steril. 2011;96(6):1306–11. 22035969 10.1016/j.fertnstert.2011.09.037 PMC3576017

[39] Lopez-Rangel E, Lewis ME. Loud and clear evidence for gene silencing by epigenetic mechanisms in autism spectrum and related neurodevelopmental disorders. Clin Genet. 2006;69(1):21–2. 16451129 10.1111/j.1399-0004.2006.00543a.x

[40] Isles AR, Wilkinson LS. Imprinted genes, cognition and behaviour. Trends Cogn Sci. 2000;4(8):309–18. 10904255 10.1016/s1364-6613(00)01504-7

[41] Turan N, Katari S, Gerson LF, Chalian R, Foster MW, Gaughan JP, et al. Inter- and intra-individual variation in allele-specific DNA methylation and gene expression in children conceived using assisted reproductive technology. PLoS Genet. 2010;6(7):e1001033. 20661447 10.1371/journal.pgen.1001033 PMC2908687

[42] Krakowiak P, Walker CK, Bremer AA, Baker AS, Ozonoff S, Hansen RL, et al. Maternal metabolic conditions and risk for autism and other neurodevelopmental disorders. Pediatrics. 2012;129(5):e1121–8. 22492772 10.1542/peds.2011-2583 PMC3340592

[43] Beversdorf DQ, Stevens HE, Margolis KG, Van de Water J. Prenatal stress and maternal immune dysregulation in autism spectrum disorders: potential points for intervention. Curr Pharm Des. 2019;25(41):4331-43 10.2174/1381612825666191119093335 PMC7100710 31742491

[44] Daniels AM, Mandell DS. Explaining differences in age at autism spectrum disorder diagnosis: a critical review. Autism. 2014;18(5):583–97. 23787411 10.1177/1362361313480277 PMC4775077

[45] Dubey P, Thakur B, Rodriguez S, Cox J, Sanchez S, Fonseca A, et al. A systematic review and meta-analysis of the association between maternal polycystic ovary syndrome and neuropsychiatric disorders in children. Transl Psychiatry. 2021;11(1):569. 34750348 10.1038/s41398-021-01699-8 PMC8575994

[46] Boulet SL, Mehta A, Kissin DM, Warner L, Kawwass JF, Jamieson DJ. Trends in use of and reproductive outcomes associated with intracytoplasmic sperm injection. JAMA. 2015;313(3):255–63. 25602996 10.1001/jama.2014.17985 PMC4343214

[47] Clarfield AM, Manor O, Nun GB, Shvarts S, Azzam ZS, Afek A, et al. Health and health care in Israel: an introduction. Lancet (London, England). 2017;389(10088):2503–13. 28495109 10.1016/S0140-6736(17)30636-0

[48] Israel HMo. In vitro fertilization (IVF) cycles in the service basket Jerusalem: legal division. 2014. [Available from: https://www.gov.il/he/Departments/policies/mr06-2014. Accessed 31 Dec 2023.

